# Laser speckle contrast imaging of perfusion in oncological clinical applications: a literature review

**DOI:** 10.2478/raon-2024-0042

**Published:** 2024-09-15

**Authors:** Rok Hren, Simona Kranjc Brezar, Urban Marhl, Gregor Sersa

**Affiliations:** Faculty of Mathematics and Physics, Ljubljana, Slovenia; Institute of Mathematics, Physics, and Mechanics, Ljubljana, Slovenia; Syreon Research Institute, Budapest, Hungary; Institute of Oncology Ljubljana, Ljubljana, Slovenia

**Keywords:** laser speckle contrast imaging (LSCI), oncology, perfusion, blood flow

## Abstract

**Background:**

Laser speckle coherence imaging (LSCI) is an emerging imaging modality that enables noninvasive visualization and assessment of tissue perfusion and microcirculation. In this article, we evaluated LSCI in imaging perfusion in clinical oncology through a systematic review of the literature.

**Methods:**

The inclusion criterion for the literature search in PubMed, Web of Science and Scopus electronic databases was the use of LSCI in clinical oncology, meaning that all animal, phantom, *ex vivo*, experimental, research and development, and purely methodological studies were excluded.

**Results:**

Thirty-six articles met the inclusion criteria. The anatomic locations of the neoplasms in the selected articles were brain (5 articles), breasts (2 articles), endocrine glands (4 articles), skin (12 articles), and the gastrointestinal tract (13 articles).

**Conclusions:**

While LSCI is emerging as an appealing imaging modality, it is crucial for more clinical sites to initiate clinical trials. A lack of standardized protocols and interpretation guidelines are posing the most significant challenge.

## Introduction

In the cancer research and treatment, the assessment of tissue perfusion and microcirculation plays a pivotal role in understanding tumor physiology, monitoring treatment responses, and determining surgical outcomes. Among the advanced visualization systems, fluorescence angiography utilizing indocyanine green (FA-ICG) has emerged as an objective tool for evaluating intraoperative perfusion.^1,2,3^ Despite its versatility, FA-ICG imaging has limitations: for example, it requires external dye injection, is constrained by pharmacokinetic factors in repeat assessments, and may potentially lead to allergic reactions to the dye.^2^ To overcome these shortcomings, novel imaging techniques have been explored for microvascular imaging.

**Figure 1.**
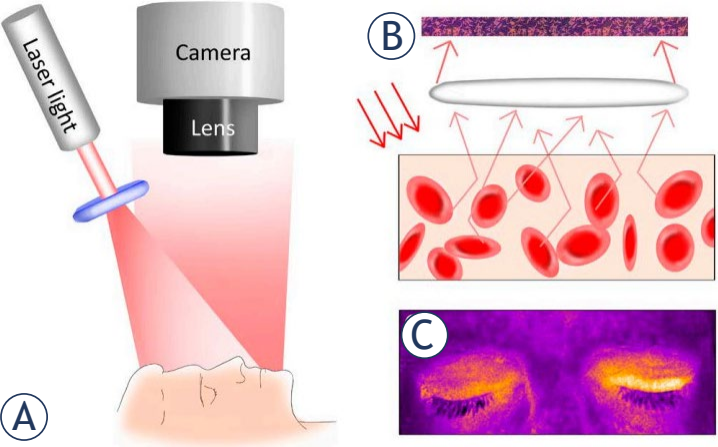
Schematic representation of the laser speckle contrast imaging (LSCI) method. **(A)** The technique relies on the interference of light backscattered from moving particles, creating distinct dark and bright areas (speckle pattern) captured by a camera. **(B)** Variations in the speckle pattern are predominantly driven by the movement of red blood cells, enabling interpretation as perfusion. **(C)** Analysis of speckle-pattern variations yields an image displayed on the monitor, where white and yellow depict areas with high perfusion, contrasting with darker areas indicating lower perfusion areas. Taken from Berggren *et al.*^19^ and reprinted with permission from the publisher.

One such modality is laser speckle contrast imaging (LSCI), a non-invasive optical imaging technique based on the unique properties of laser light to visualize blood flow and tissue perfusion in real-time.^4,5^ At the core of LSCI lies the phenomenon of capturing the dynamic interference pattern, known as speckle, created when coherent laser light interacts with moving particles such as red blood cells, generating a real-time 2D color heatmap of blood flow (Figure 1).^6^ By analyzing the temporal fluctuations in the speckle pattern, LSCI can quantitatively assess blood flow velocity, perfusion dynamics, and tissue microcirculation with high spatial and temporal resolution.

LSCI is a versatile modality with its applicability ranging from material science^7^ to notable applications in medical therapeutic segments.^8^ LSCI has aided, among others, in studying retinal blood flow^9^, cardiovascular diseases^10,11^ and organ perfusion^6,12^, while demonstrating potential as a valuable tool for assessing burns^13,14,15^ and wound healing processes^16,17,18^, and monitoring perfusion during reconstructive surgery^19^ and neurosurgery.^20,21,22,23,24,25,26^ The value of LSCI in quantifying blood flow dynamics within clinical oncology remains unclear, and to that end, we systematically reviewed the literature with a specific focus on studies in which LSCI was conducted on patients in a clinical oncology setting.

## Methods

Authors conducted jointly—to minimize potential bias—a comprehensive literature search on April 16, 2024, through PubMed, Web of Science and Scopus electronic databases using the following search terms: “laser speckle coherence imaging tumors”, “laser speckle coherence imaging cancer”, “laser speckle coherence imaging carcinoma”, “laser speckle coherence imaging anastomosis”, and “laser speckle coherence imaging thyroid”. No restrictions on publication date or language were imposed. The inclusion criterion was the application of LSCI in a clinical oncological setting, meaning that all animal and phantom, *ex vivo*, experimental, research and development, and purely methodological studies were excluded. Special care was taken to remove duplicates across databases and studies; for example, if the study was first published in proceedings and later in a journal, the proceedings article was considered a non-primary publication and therefore excluded. Studies were categorized with respect to the anatomical location of the tumors.

## Results

In total, 309 articles were found to be of interest in the PubMed, Web of Science and Scopus databases. After excluding duplicates and applying the exclusion criteria, first considering the title and abstract and then, if necessary, reading the entire article, 36 articles were identified for further analysis. The anatomical locations of tumors in the selected articles were as follows: brain (5 articles), breasts (2 articles), endocrine glands (4 articles), skin (12 articles), and the gastrointestinal (GI) tract (13 articles).

### Brain

Parthasarathy *et al*.^21^ made a pioneering effort in the evaluation of perfusion in clinical oncology using LSCI. Their pilot study focused on imaging cerebral blood flow either before (1 patient) or after (2 patients) tumor resections, across various cortical regions. The same group continued research on larger patient groups (10 and 8, respectively), demonstrating the feasibility of using LSCI to monitor blood flow during neurosurgery.^22,27^ Despite these promising outcomes, their research output ceased after 2017.

**Table 1.** Included articles reporting the use of laser speckle contrast imaging (LSCI) to quantify perfusion in clinical applications in oncology

**Reference**	**Year of publication**	**Number of patients**	**Oncologic setting**
***Brain***			
**Parthasarathy *et al.*^21^**	2010	3	Tumor resection
**Richards *et al.*^22^**	2014	10	Tumor resection
**Richards *et al*.^27^**	2017	8	Tumor resection
**Klijn *et al*.^25^**	2013	8	Tumor resection
**Ideguchi *et al*.^28^**	2017	12	Tumor resection
***Breasts***			
**Tesselaar *et al.*^29^**	2017	15	Adjuvant radiotherapy for stage I-II breast cancer
**Zötterman *et al.*^30^**	2020	23	Deep inferior epigastric artery perforator (DIEP) flap surgery
***Endocrine glands***			
**de Paula *et al.*^31^**	2021	42	Non-functioning adrenal incidentaloma
**Mannoh *et al.*^32^**	2017	28	Thyroidectomy/parathyroidectomy
**Mannoh *et al.*^33^**	2021	72	Thyroidectomy
**Mannoh *et al.*^34^**	2023	21	Thyroidectomy/parathyroidectomy
***Skin***			
**Tchvialeva *et al.*^35^**	2012	214 lesions	Malignant melanoma, squamous cell carcinoma, basal cell carcinoma, melanocytic nevus, seborrheic keratosis
**Reyal *et al.*^36^**	2012	12	Basal cell carcinoma
**Zhang *et al.*^37^**	2019	12 (total 143)	Facial nerve palsy due to nerve tumor (also including other etiology)
**Zieger *et al.*^38^**	2021	9	Basal cell carcinoma
**Tenland *et al.*^39^**	2019	13	Oculoplastic reconstructive surgery (tarsoconjunctival flaps)
**Berggren *et al.*^40^**	2019	9	Oculoplastic reconstructive surgery (tarsoconjunctival flaps)
**Tenland *et al.*^41^**	2021	12	Oculoplastic reconstructive surgery after squamous cell carcinoma, basal cell carcinoma, and intradermal nevus
**Berggren *et al.*^42^**	2021	7	Oculoplastic reconstructive surgery after squamous cell carcinoma and basal cell carcinoma
**Berggren *et al.*^43^**	2021	7	Oculoplastic reconstructive surgery after squamous cell carcinoma and basal cell carcinoma
**Berggren *et al.*^44^**	2021	1	Oculoplastic reconstructive surgery
**Berggren *et al.*^45^**	2022	7	Oculoplastic reconstructive surgery after squamous cell carcinoma and basal cell carcinoma
**Stridh *et al.*^46^**	2024	1	Cutaneous angio-sarcoma
***Gastrointestinal tract (open surgical setting)***			
**Eriksson *et al.*^47^**	2014	10	Liver resection
**Milstein *et al.*^48^**	2016	11	Esophagectomy
**Ambrus *et al.*^49^**	2017	45	Esophagectomy
**Ambrus *et al.*^50^**	2017	25	Ivor-Lewis esophagectomy
**Di Maria *et al.*^51^**	2017	2	Colorectal resection
**Jansen *et al.*^52^**	2018	26	Esophagectomy
**Kojima *et al.*^53^**	2019	8	Colorectal resection
**Kaneko *et al.*^54^**	2020	36	Colorectal resection (34 due to colorectal carcinoma)
***Gastrointestinal tract (laparoscopic/thoracoscopic setting)***			
**Heeman *et al.*^55^**	2019	10	Colorectal resection
**Kojima *et al.*^56^**	2020	27	Colorectal resection
**Slooter *et al.*^57^**	2020	24	Esophagectomy
**Heeman *et al.*^58^**	2023	67	Hemicolectomy and sigmoid resection
**Nwaiwu *et al.*^59^**	2023	40	Colectomy, also non-oncological interventions (Roux-en-Y gastric bypass and sleeve gastrectomy)

Another research group^25^ highlighted the potential of LSCI for functional brain mapping during awake craniotomy for tumor removal. They observed a strong correlation between cortical microvascular blood flow, as determined by LSCI, and electrocortical stimulation mapping. Additionally, Ideguchi *et al.*^28^ emphasized the capability of LSCI for noninvasive and rapid intraoperative real-time recognition of mass lesion-related vasculature, which could be crucial in mitigating ischemic complications and complementing neurophysiological monitoring.

### Breasts

Tesselaar *et al.*^29^ conducted a study exploring the relationship between radiation exposure and changes in microvascular perfusion in 15 women undergoing adjuvant radiation therapy for stage I-II breast cancer. Their findings suggested that LSCI holds promise as a useful tool for objectively assessing radiation-induced microvascular changes in the skin, even before visible changes occur, thereby aiding in the earlier prediction of potential severe reactions.

In another prospective clinical pilot study conducted across two centers^30^, LSCI was employed in 23 women undergoing primary, secondary, or tertiary deep inferior epigastric artery perforator (DIEP) procedures, either unilateral or bilateral. Researchers used laser speckle patterns to calculate perfusion values in arbitrary units (PU), reflecting the concentration and mean velocity of red blood cells. Categorizing patients into high (> 30) and low (< 30) PU, they found that all flaps with perfusion < 30 PU immediately after surgery had postoperative complications, necessitating revision in 4 women. These results suggest potential utility of LSCI for early detection of flap necrosis, aiding surgeons in identifying viable parts of the flaps. Traditionally, assessment of flap viability relies on subjective methods like skin color, flap temperature, capillary refill time, and dermal edge bleeding.

### Endocrine glands

Endothelial reactivity^60,61^ was evaluated by LSCI in patients with mostly benign non-functioning adrenal incidentaloma.^31^. Mannoh *et al.*^32^ used LSCI to assess parathyroid viability post-thyroidectomy in 20 patients, achieving an accuracy of 91.5% in distinguishing between well vascularized (n = 32) and compromised (n = 27) parathyroid glands compared to visual assessment by an experienced surgeon. Ability to detect vascular compromise with LSCI was further validated in parathyroidectomies in 8 patients, showing that this technique could identify parathyroid gland devascularization before it became visually apparent to the surgeon. LSCI demonstrated promise as a real-time, contrast-free, objective method to mitigate hypoparathyroidism after thyroid surgery.

**Figure 2.**
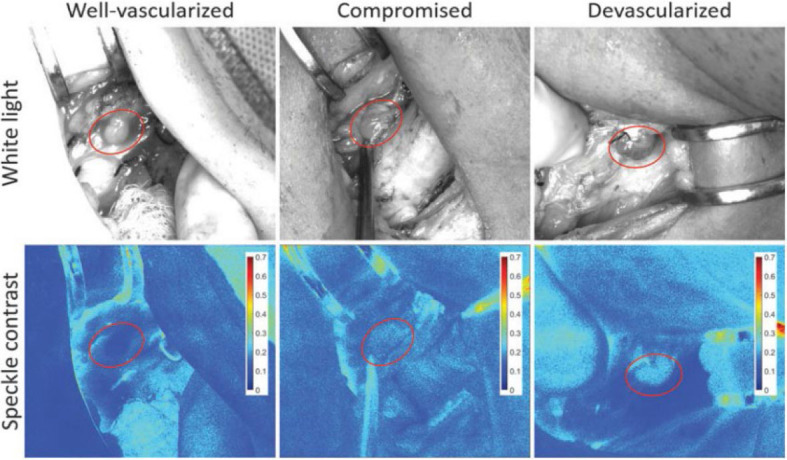
Speckle contrast demonstrates lower values for well-vascularized parathyroid glands. Lower speckle contrast values indicate greater blood flow due to more blurring of the speckle pattern, while higher contrast values indicate less blood flow. The top row displays representative white light images, and the bottom row shows speckle contrast images of a well-vascularized (left), a compromised (middle), and a devascularized (right) parathyroid gland, with parathyroid glands marked with ellipses. The corresponding speckle contrast values were 0.11, 0.18, and 0.21, respectively. Taken from Mannoh *et al*.^33^ and reprinted with permission from the publisher.

Subsequently, Mannoh *et al.*^33^ expanded their research, enrolling 72 patients who underwent thyroidectomy. They established an intraoperative speckle contrast threshold of 0.186 to distinguish between normoparathyroid and hypoparathyroid groups with 87.5% sensitivity and 84.4% specificity. This threshold served as an indicator of adequate parathyroid vascularization, with glands below the value of 0.186 considered adequately perfused (Figure 2).

Additionally, Mannoh *et al*.^34^ combined LSCI with ICG angiography in 21 patients undergoing thyroidectomy or parathyroidectomy. While both modalities offered similar information on parathyroid gland blood flow, they suggested advantages of LSCI, including lower costs, non-invasiveness, absence of contraindications, and compatibility with near-infrared autofluorescence (NIRAF) detection, which has recently emerged as a reliable technique for intraoperative parathyroid gland localization or confirmation.^62,63,64^

### Skin

Tchvialeva *et al.*^35^ applied LSCI to differentiate among 214 skin lesions, encompassing the three major types of skin cancers (malignant melanoma, squamous cell carcinomas, and basal cell carcinomas – BCCs), and two benign conditions (melanocytic nevus and seborrheic keratoses). In another early clinical study, LSCI was used to demonstrate that post-occlusive reactive hyperemia could occur in BCC as well.^36^ Zhang *et al.*^37^ explored differences in facial microvascular perfusion between ipsilateral and contralateral sides in patients with facial nerve palsy (FNP), observing significant decreases on the ipsilateral side, which improved after treatment. In their feasibility study, Zieger *et al.*^38^ introduced a compact handheld LSCI device, affirming its reliability in assessing BCC.

**Figure 3.**
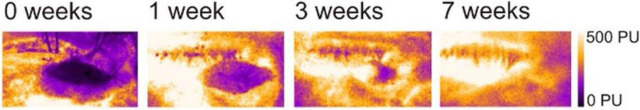
Representative examples of laser speckle contrast images, showing the blood perfusion in the free skin grafts, immediately postoperatively (0 weeks), and at follow-up after 1, 3, and 7 weeks. It can be seen that reperfusion occurred simultaneously in the center and periphery of the graft, and that complete reperfusion was achieved after 7 weeks. Taken from Berggren *et al.*^43^ and reprinted with permission from the publisher.

In oculoplastics, Tenland *et al.*^39^ and Berggren *et al.*^40^ conducted studies using LSCI to monitor perfusion in patients with lower eyelid defects after post-tumor surgery large enough to require a tarsoconjunctival graft. Building on their initial work, the group continued research of employing LSCI in various oculoplastic reconstructive surgery procedures. First, Tenland *et al.*^41^ monitored perfusion using LSCI in a study in which free bilamellar eyelid grafts appeared to be an excellent alternative to the tarsoconjunctival flap procedure in the reconstruction of both upper and lower eyelid defects. Next, Berggren *et al.*^42^ noted rapid revascularization of H-plasty procedure flaps within a week postoperatively, attributing it to the pre-existing vascular network of the flap pedicle, rather than significant angiogenesis. In another study, Berggren *et al.*^43^ demonstrated complete reperfusion of skin grafts in the periorbital area after 7 weeks (Figure 3). Berggren *et al.*^44^ also presented a case illustrating nearly complete restoration of reperfusion in a rotational full-thickness lower eyelid flap within 5 weeks. Finally, they assessed blood perfusion in glabellar flaps, finding rapid reperfusion.^45^ These convincing findings suggest that perioperative LSCI monitoring of perfusion in human periocular flaps and during oculoplastic reconstructive surgery offers an attractive imaging modality for routine clinical use. Not surprisingly, Stridh *et al.*^46^ recently conducted a pilot study comprehensively combining LSCI with two other emerging non-invasive medical imaging modalities, hyperspectral imaging^65,66,67^ and photoacoustic imaging^68^ to monitor not only blood perfusion but also oxygen saturation and the molecular composition of the tissue.

### Gastrointestinal tract (open surgical setting)

The majority of clinical oncology studies with intraoperative LSCI were conducted in an open surgical setting, which we will review first. In an initial pilot clinical study, Eriksson *et al*.^47^ assessed liver blood perfusion by occluding the portal vein and hepatic artery in ten consecutive patients undergoing liver resection for colorectal liver metastases. This early effort was followed by Milstein *et al.*^48^, who evaluated microvascular blood flow during esophagectomy, affirming that intraoperative LSCI offered a non-contact, non-invasive approach for real-time analysis of potential anastomotic leakage without requiring a contrast medium. This finding was subsequently corroborated by Ambrus *et al.* who first performed gastric microvascular perfusion measurements during esophagectomy in 45 patients^49^ and later used LSCI in Ivor-Lewis esophagectomy in 25 patients.^50^

Di Maria *et al.*^51^ explored the feasibility of LSCI in 2 patients undergoing colorectal surgery, while Jansen *et al*.^52^ investigated the impact of thoracic epidural anesthesia during esophagectomy, once again demonstrating that LSCI could detect subtle changes in gastric microvascular perfusion in realtime. Another group conducted an additional feasibility study of intraoperative LSCI in 8 patients undergoing colorectal surgery.^53^ Kaneko *et al.*^54^ further expanded on these feasibility studies by enrolling 36 patients undergoing colorectal resection, 34 of whom had colorectal carcinoma, aiming to compare demarcation lines determined by LSCI with transection lines where marginal vessels were divided. They found that 58.3% (21/36) of demarcation lines matched transection lines, with a median distance of 0.0 mm (0.0–12.1 mm) between the demarcation line determined by LSCI and the transection line.

### Gastrointestinal tract (laparoscopic/thoracoscopic setting)

**Figure 4.**
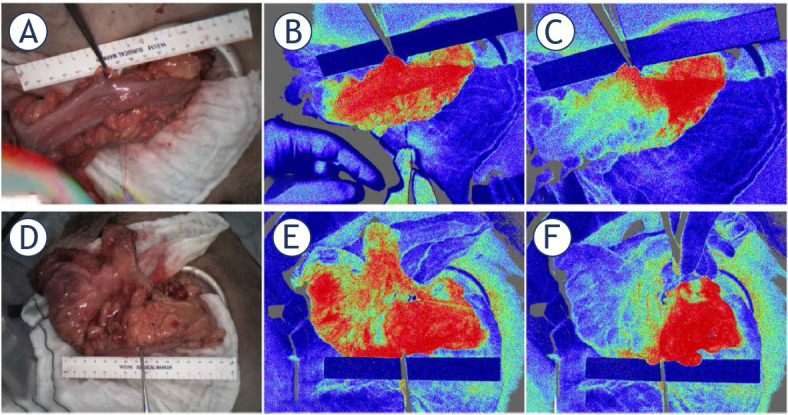
Typical laser speckle images in two patients. High-resolution laser speckle contrast imaging (LSCI) can indicate the bowel demarcation line at the point of ligation of the marginal vessels. **(A)** Normal color image before ligating the marginal vessels. **(B)** LSCI image before ligating the marginal vessels. **(C)** LSCI image after ligating the marginal vessels. Taken from Kojima *et al.*^56^ and reprinted with permission from the publisher.

Heeman *et al.*^55^ reported the first intraabdominal application combining a standard laparoscopic surgical setup with LSCI in 10 patients, enabling imaging of intestinal blood flow during a vascular occlusion test. Their findings were corroborated by Kojima *et al*.^56^ in a study involving 27 patients (Figure 4). Slooter *et al.*^57^ systematically compared four different emerging optical modalities, highlighting the clinical utility of FA-ICG as the most promising. Recently, Heeman *et al.*^58^ tested a commercial LSCI system in the oncological clinical setting, noting that the system was “non-disruptive of the surgical procedure with an average added surgical time of only 2.5 min and no change in surgical equipment”. They also observed a potential clinical benefit of the LSCI system, with 17% of operating surgeons altering anastomosis locations based on perfusion assessments. Nwaiwu *et al.*^58^ evaluated another commercial intraoperative system combining LSCI and FA-ICG in mostly non-oncological patients, demonstrating that LSCI identified the same perfusion boundaries as FA-ICG, with anastomoses and gastric remnants appearing well perfused.

## Discussion

Based on this literature review, several advantages of LSCI emerge, including its non-invasive and non-contact nature, short acquisition time, high spatial and temporal resolution, low cost of equipment, and simplicity of operation. In the oncological clinical setting, LSCI holds particular promise for assessing skin flap perfusion post-oculoplastic reconstructive surgery and anastomotic perfusion during gastrointestinal reconstruction. While LSCI offers numerous advantages in imaging blood flow dynamics, it is essential to recognize its limitations.

### Limited penetration depth

One of the obvious limitations of LSCI in clinical oncology and medical applications, in general, is its restricted penetration depth. LSCI relies on detecting motion contrast generated by moving red blood cells, limiting its applicability to superficial structures. Tumors and lesions located in deeper anatomical locations, such as within organs or soft tissues, may not be adequately visualized due to this limitation, hindering comprehensive evaluation and monitoring of oncological conditions. However, studies like that of Stridh *et al.*^46^ demonstrate that PAI as a complementary imaging technique can overcome this limitation. Another possibility to potentially consider is the use of optical clearance techniques^69^ to enhance tissue transparency and improve light penetration depth.

### Motion artifacts

LSCI is susceptible to motion artifacts, which can arise from either involuntary movement of the subject or vibrations in the imaging setup. These artifacts can lead to image distortions and reduced image quality, compromising the accuracy and reliability of LSCI in clinical oncology. To address this, advanced post-processing algorithms are necessary to improve image quality. Since motion artifacts are well-known sources of artifacts in LSCI, they have been extensively researched. One possibility is to implement motion compensation techniques, such as image stabilization algorithms^70^ or gating strategies^71^, which can mitigate the effects of motion artifacts in LSCI. By minimizing motion-induced distortions in the speckle pattern, these techniques improve the accuracy and reliability of blood flow measurements.

### Inherent speckle noise

The presence of inherent speckle noise in LSCI images can compromise the accuracy and reliability of blood flow measurements, particularly in low-flow regions or under conditions of low contrast. Speckle noise can obscure subtle flow changes and restrict the sensitivity of LSCI in detecting small-scale perfusion variations. Advanced noise reduction algorithms^72^ offer a solution by effectively suppressing speckle noise and enhancing the signal-to-noise ratio. These algorithms filter out unwanted noise components while retaining relevant flow information, thereby improving the sensitivity and specificity of LSCI in detecting perfusion changes, even in challenging imaging conditions.

### Lack of standardized protocols and interpretation

A significant limitation of LSCI in clinical oncology is the lack of standardized protocols and interpretation guidelines. Varying acquisition settings, image processing algorithms, or interpretation methodologies across different centers can yield inconsistent and non-comparable results. Establishing standardized protocols and guidelines tailored to oncology applications would enhance the accuracy and reproducibility of LSCI findings.

Despite its potential, the clinical integration of LSCI faces obstacles, including the standardization of imaging protocols, validation of its utility in large-scale clinical trials, and integration into existing surgical workflows. Addressing these limitations requires advancements in technology, algorithm refinement, and increased participation of clinical sites in conducting trials. Overcoming these challenges is essential for realizing the full potential of LSCI in clinical oncology; it is worth noting that other biomedical optical imaging techniques^65,66,67,73,74,75,76,77,78,79,80^ are likely to encounter similar challenges in the future.
