# Laser speckle contrast imaging of perfusion in oncological clinical applications: a literature review

**DOI:** 10.2478/raon-2025-0045

**Published:** 2025-08-06

**Authors:** Rok Hren, Simona Kranjc Brezar, Urban Marhl, Gregor Sersa

**Affiliations:** Faculty of Mathematics and Physics, Ljubljana, Slovenia; Institute of Mathematics, Physics, and Mechanics, Ljubljana, Slovenia; Syreon Research Institute, Budapest, Hungary; Institute of Oncology Ljubljana, Ljubljana, Slovenia

In the original article, the first sentence in the Abstract section is incorrect. Instead of »Laser speckle coherence imaging (LSCI) is an emerging imaging modality that enables noninvasive visualization and assessment of tissue perfusion and microcirculation.« it should be »Laser speckle contrast imaging (LSCI) is an emerging imaging modality that enables noninvasive visualization and assessment of tissue perfusion and microcirculation.«

